# Impact of an Augmented Reality Navigation System (SIRIO) on Bone Percutaneous Procedures: A Comparative Analysis with Standard CT-Guided Technique

**DOI:** 10.3390/curroncol28030163

**Published:** 2021-05-08

**Authors:** Eliodoro Faiella, Gennaro Castiello, Caterina Bernetti, Giuseppina Pacella, Carlo Altomare, Flavio Andresciani, Bruno Beomonte Zobel, Rosario Francesco Grasso

**Affiliations:** Department of Diagnostic and Interventional Radiology, Campus Bio-Medico of Rome, Via Alvaro del Portillo, 200, 00128 Rome, Italy; g.castiello@unicampus.it (G.C.); c.bernetti@unicampus.it (C.B.); g.pacella@unicampus.it (G.P.); c.altomare@unicampus.it (C.A.); f.andresciani@unicampus.it (F.A.); b.zobel@unicampus.it (B.B.Z.); r.grasso@unicampus.it (R.F.G.)

**Keywords:** optical tracking, Computed Tomography, bone neoplasm, biopsy, ablation techniques

## Abstract

(1) Background: The purpose of this study is to evaluate the impact of an augmented reality navigation system (SIRIO) for percutaneous biopsies and ablative treatments on bone lesions, compared to a standard CT-guided technique. (2) Methods: Bioptic and ablative procedures on bone lesions were retrospectively analyzed. All procedures were divided into SIRIO and Non-SIRIO groups and in <2 cm and >2 cm groups. Number of CT-scans, procedural time and patient’s radiation dose were reported for each group. Diagnostic accuracy was obtained for bioptic procedures. (3) Results: One-hundred-ninety-three procedures were evaluated: 142 biopsies and 51 ablations. Seventy-four biopsy procedures were performed using SIRIO and 68 under standard CT-guidance; 27 ablative procedures were performed using SIRIO and 24 under standard CT-guidance. A statistically significant reduction in the number of CT-scans, procedural time and radiation dose was observed for percutaneous procedures performed using SIRIO, in both <2 cm and >2 cm groups. The greatest difference in all variables examined was found for procedures performed on lesions <2 cm. Higher diagnostic accuracy was found for all SIRIO-assisted biopsies. No major or minor complications occurred in any procedures. (4) Conclusions: The use of SIRIO significantly reduces the number of CT-scans, procedural time and patient’s radiation dose in CT-guided percutaneous bone procedures, particularly for lesions <2 cm. An improvement in diagnostic accuracy was also achieved in SIRIO-assisted biopsies.

## 1. Introduction

Interventional radiology plays an important role in the management of bone lesions. Percutaneous procedures of biopsy and ablation are widely validated in the diagnostic and therapeutic course of patients suffering from diseases involving the skeletal system [1,2]. Computed Tomography (CT) is the most common imaging technique used to guide percutaneous procedures on bone lesions, although CT guidance is limited by the high amount of radiation dose administered to patients and by the lack of real-time visualization [3]. Recently, different navigation systems have been introduced to assist imaging-guided procedures, improving accuracy and precision in reaching the target lesion, thus reducing procedural time and radiation dose [4]. Such systems allow electromagnetic (EM) [5,6,7], optical [8,9] or hybrid [10,11] tracking and real-time visualization of the devices used during interventional procedures. EM navigation systems, based on EM field generators and EM sensors, have been applied to perform procedures such as thermal ablations of hepatic tumors [12,13] with a reduction in radiation exposure for patients [12], and for interventions in other surgical fields [14,15,16]. Flat-panel cone beam CT navigation systems based on fusion imaging have also been proposed in the last few years to assist procedures such as percutaneous biopsies on bones [17,18] or lungs [19], and are considered an effective method that simplifies needle path planning and shortens procedure times. Laser tracking systems [20] and navigational robots [21] have also been employed for navigation during image-guided interventions. Optical navigation systems, based on video cameras or sensors detecting infrared light, allow tracking of the position of the operational devices. These have been used to reach difficult-to-treat lesions in organs such as the liver [22] and were validated to perform percutaneous low-dose CT-guided lung biopsies [23]. Each of these systems has substantial advantages over traditional guidance systems, however do present certain limitations specific to each system, such as the lack of availability of devices compatible with EM fields for all types of percutaneous procedures, the need for an unhindered path between the camera and the instrument layout for optical systems, added cost and setup time for laser guidance, large size of instrumentation for robotic assisted systems and radiation exposure for operators while using flat-panel cone beam CT navigation systems [4]. In this paper, we evaluate the use of an optical-based navigation system (SIRIO, MASMEC S.p.A., Modugno, Bari, Italy) for percutaneous biopsies and ablative treatments on bone lesions. Data obtained from SIRIO-guided procedures were compared to those obtained from procedures performed under conventional CT-guidance.

## 2. Materials and Methods

### 2.1. Patients and Groups

**Figure 1:**
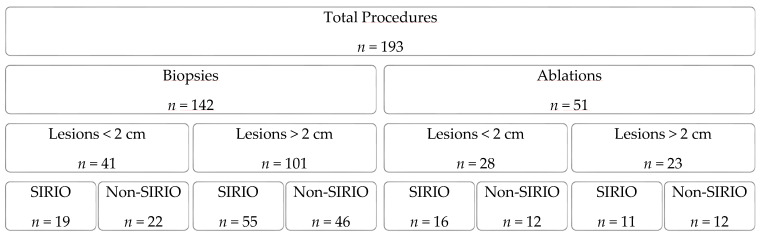
This diagram shows the subdivision of the procedures into the different studied groups, specifying the number of procedures for each group.

This retrospective study was approved by the local Institutional Review Board. A total of 193 procedures on bone lesions, performed from January 2006 to July 2019 at the Department of Interventional Radiology of our institution, were consecutively enrolled. Inclusion criteria were: age > 16 years, bone lesions suspected to be malignant on MRI, CT and/or PET-CT images with clinical indication to undergo percutaneous biopsy, biopsy-proven bone lesions with clinical indication to undergo percutaneous ablative treatment, and good patient compliance. Exclusion criteria were: contraindications for percutaneous interventions (e.g., abnormal coagulation state), refusal to provide written informed consent, and no patient compliance. All procedures were divided into biopsy and ablation groups and randomly assigned to SIRIO group or to standard CT-guidance group (non-SIRIO). Each group was also divided into two subgroups based on lesion size, according to a cut-off of 2 cm (<2 cm; >2 cm) (Figure 1). Patients’ lesions characteristics are summarized in Table 1 and Table 2. For each patient, medical records, previous imaging exams, laboratory studies, and lesion-related pathological information (when available) were carefully evaluated. In general, for biopsies on lesions <2 cm, there were no important differences between the SIRIO and non-SIRIO groups in terms of age (61.4 years; 56.8 years; *p* = 0.37), gender (7 ♂, 12 ♀; 10 ♂, 12 ♀; *p* = 0.576) and lesion type (11 non-malignant, 8 malignant; 13 non-malignant, 9 malignant; *p* = 0.938). In general, for biopsies on lesions >2 cm, there were no important differences between the SIRIO and non-SIRIO groups in terms of age (60.9 years; 61.3 years; *p* = 0.481), gender (22 ♂, 33 ♀; 15 ♂, 31 ♀; *p* = 0.443) and lesion type (23 non-malignant, 32 malignant; 18 non-malignant, 28 malignant; *p* = 0.784). In general, for ablations on lesions <2 cm, there were no important differences between the SIRIO and non-SIRIO groups in terms of age (29.4 years; 29.7 years; *p* = 0.491), gender (12 ♂, 4 ♀; 6 ♂, 6 ♀; *p* = 0.172) and lesion type (11 non-malignant, 5 malignant; 9 non-malignant, 3 malignant; *p* = 0.717). In general, for ablations on lesions > 2 cm, there were no important differences between the SIRIO and non-SIRIO groups in terms of age (65.9 years; 55.5 years; *p* = 0.296), gender (5 ♂, 6 ♀; 5 ♂, 7 ♀; *p* = 0.855) and lesion type (1 non-malignant, 10 malignant; 2 non-malignant, 10 malignant; *p* = 0.59).

### 2.2. Procedures

All biopsy and ablation procedures were performed in a dedicated CT-room by four experienced interventional radiologists, either under SIRIO or standard CT-guidance. During the procedures, patients were lying in a position chosen to guarantee the shortest distance between the lesion and the skin. All procedures were performed using a 64-slice multidetector Computed Tomography (Somaton Sensation 64, Siemens Healthineers, Erlangen, Germany) with the following exposure parameters: 64 × 0.6 mm^2^ detector configuration, pitch 1.4, table speed 0.81 mm/rotation, 0.33-s gantry rotation, tube voltage of 120 kV, tube real-time dose modulation (CARE Dose4D^TM^) of 80–250 mAs, slice thickness 2.3 mm, reconstruction interval 1 mm.

#### 2.2.1. Biopsies

In the biopsy procedures, different types of biopsy needles (ranging from 11 G and 18 G) were used to reach and sample bone lesions. Procedures were performed under local anesthesia and optional mild sedation.

#### 2.2.2. Ablations

Ablation procedures were performed using three different techniques chosen depending on lesion site and dimension: radiofrequency ablation (RFA), cryoablation (CA) and microwave ablation (MWA) [24]. In selected cases, protective measures such as temperature monitoring, fluid dissection or CO_2_ dissection were adopted to avoid unintentional ablation of nearby non-target organs. Deep sedation, general anesthesia, nerve block or spinal anesthesia were performed during the procedures.

### 2.3. SIRIO Augmented Reality Navigation System

**Figure 2:**
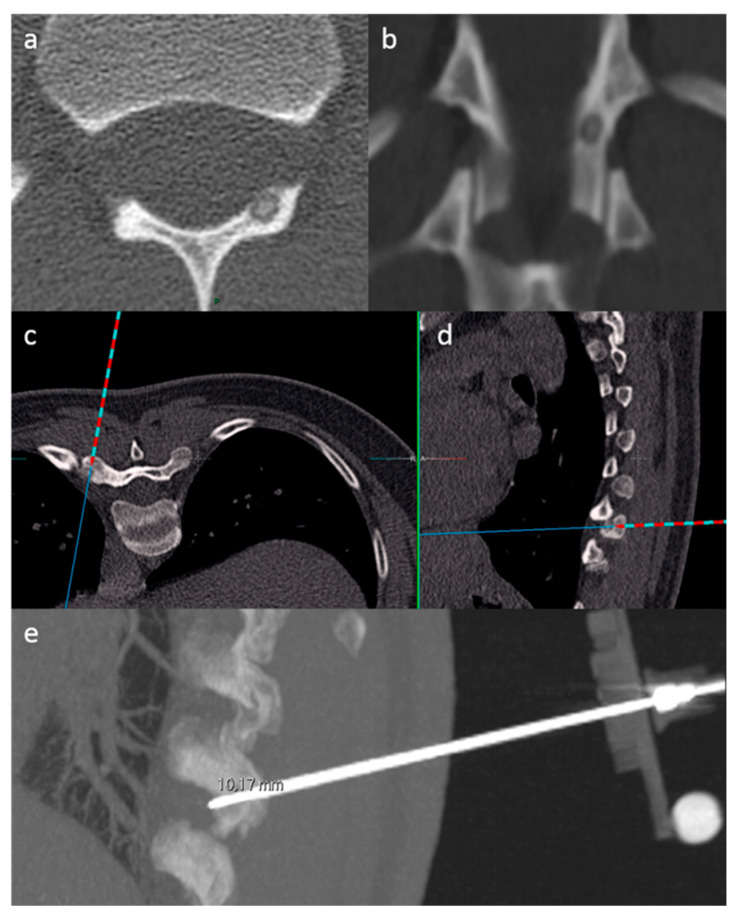
SIRIO-assisted needle positioning within an osteoid osteoma of the left lamina of a thoracic vertebra, in order to perform a biopsy procedure. Figures in (**a**,**b**) show CT images of the osteoid osteoma. Axial (**c**) and sagittal (**d**) reconstructions of the virtual 3D model show the needle trajectory to reach the lesion. The sagittal reconstruction in (**e**) confirms the correct positioning of the needle within the lesion; notice the needle tool with a passive sphere included in the CT scan (lower right).

**Figure 3:**
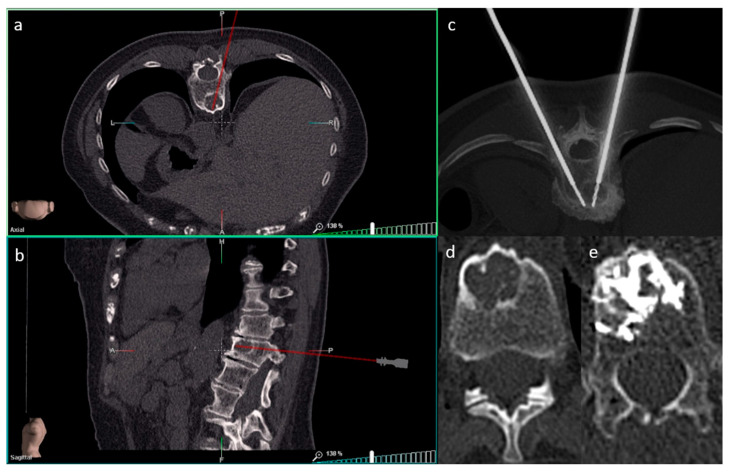
SIRIO-assisted needle positioning within a breast cancer metastasis of a lumbar vertebral body in order to perform an ablation procedure followed by cementoplasty. Axial (**a**) and sagittal (**b**) reconstructions of the virtual 3D model show the needle trajectory to the lesion. The axial reconstruction in (**c**) confirms the correct positioning of the needles within the lesion. The axial reconstructions in (**d**,**e**) show the bone lesion before (**d**) and after (**e**) the procedure with optimal final distribution of cement within the lesion (**e**).

SIRIO is an intraoperative augmented reality navigation system that reconstructs a 3D model from formerly acquired CT images using a semiautomatic algorithm [3,23]. The system is formed by a patient tool (PT), a needle tool (NT), a visualization/elaboration unit (VU) and an infrared optical sensor (OS) placed on the CT room ceiling. The PT is positioned near the target area, in a stable anatomical district and preferably above a bony structure to minimize tool movements during the procedure. The NT is positioned on the proximal end of the biopsy needle or ablation instrument. The PT and the NT have four fixed passive spheres capable of reflecting infrared light which is detected by the OS. A set of DICOM images acquired from a preliminary CT scan are then analyzed by a proprietary reconstruction algorithm that creates a 3D virtual model of the patient’s anatomical target area, which is spatially bounded to the real patient’s anatomical target area through an automatic calibration procedure using the PT as reference. During the procedure, two planar projections of the virtual model (axial and sagittal) are shown on the VU, dynamically calculated and updated on the screen according to the actual NT position and orientation. SIRIO is able to track the position of the NT, allowing virtual instrument movements to be reproduced inside the patient’s target district, using the reformatted 3D model. Therefore, the operational instrument can be advanced inside the lesion with extreme accuracy (Figure 2 and Figure 3). 

### 2.4. Data Collection

Patients’ demographics and procedure-related data were collected. The number of CT scans for each procedure was obtained by the data stored on the local picture archiving and communication system (PACS). Radiation dose was estimated considering total dose-length product (TDLP; mGy*cm). TDLP is the product of CTDI_Vol_ and scan length (in centimeters), a measure of the total amount of radiation used to perform any CT examination and representing a valid tool for comparison between the overall radiation dose in the studied groups [25]. Procedural time was estimated by recording the difference between the clock reading on the CT scanogram and on the last CT acquisition showing the used instrument within the target lesion, not considering the CT scan acquired at the end of the procedures, after the removal of the biopsy or ablation instruments, performed to rule out post-procedural complications.

### 2.5. Statistics

The differences in terms of number of CT scans, patient radiation exposure and procedural time were analyzed between the procedures performed with SIRIO and without SIRIO in both dimensional groups (<2 cm and >2 cm) and in both procedural groups (biopsies and ablations). A comparison between SIRIO and non-SIRIO groups was performed for each variable with the t test (*p* < 0.01) and was represented by boxplot graphs. For biopsy procedures sensitivity, specificity and diagnostic accuracy were calculated in both SIRIO and non-SIRIO groups. All the statistics were elaborated using IBM SPSS Statistics for Windows, version 26 (IBM Corp., Armonk, New York, NY, USA).

## 3. Results

**Figure 4:**
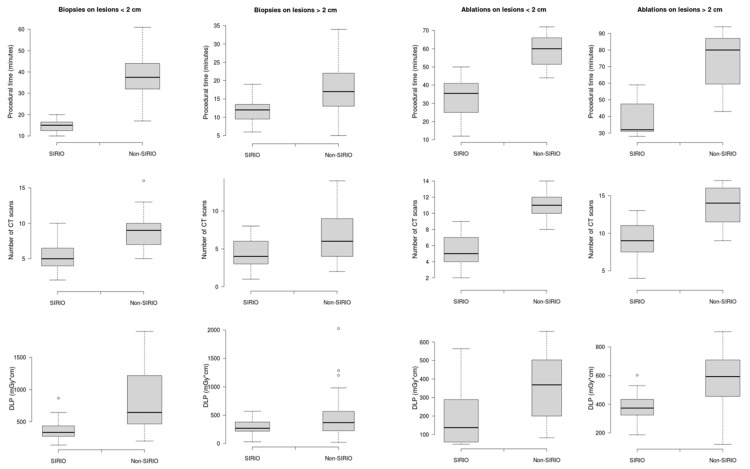
Boxplot graphs showing the differences between procedures performed using SIRIO and standard CT-guidance (non-SIRIO), for all the considered variables (procedural time, number of CT scans and radiation dose) and in both dimensional groups (<2 cm; >2 cm).

A total of 193 procedures were performed: 142 biopsies and 51 ablations. Seventy-four biopsy procedures were performed using SIRIO and 68 under standard CT-guidance; 27 ablative procedures were performed using SIRIO and 24 under standard CT-guidance. Mean values with standard deviations related to each variable and for each group are shown in Table 3. In biopsies on lesions <2 cm procedural time, number of CT-scans and radiation dose were lower in the SIRIO group than in the non-SIRIO group, with a mean difference of 23.5 min (95% CI, 18–28.9; *p* < 0.05), four CT-scans (95% CI, 2.4–5.6; *p* < 0.05) and 459.5 mGy*cm (95% CI, 225.6—693.4; *p* < 0.05). The same variables were also lower for biopsies in lesions >2 cm in the SIRIO group compared to the non-SIRIO group, with a mean difference of 2.3 min (95% CI, 1–3.6; *p* < 0.05), two CT-scans (95% CI, 1–3; *p* < 0.05) and 132.1 mGy*cm (95% CI, 25.6–238.6; *p* < 0.05). In ablations on lesions < 2 cm procedural time, number of CT-scans and radiation dose were lower in the SIRIO group than in the non-SIRIO group, with a mean difference of 25.6 min (95% CI, 17.6–33.6; *p* < 0.05), six CT-scans (95% CI, 4.4–7.6; *p* < 0.05) and 170.1 mGy*cm (95% CI, 29.6–310.6; *p* < 0.05). The same variables were also lower for ablations in lesions >2 cm in the SIRIO group compared to the non-SIRIO group, with a mean difference of 35.6 min (95% CI, 22.6–48.7; *p* < 0.05), five CT-scans (95% CI, 2.4–7.6; *p* < 0.05) and 172.7 mGy*cm (95% CI, 7.5–337.9; *p* < 0.05). Boxplots of the three variables, for each group, are shown in Figure 4. SIRIO-guided and standard CT-guided biopsies showed a diagnostic accuracy of 93.4% vs. 89.8% respectively in lesions <2 cm, and of 96.8% vs. 95.6% respectively in lesions >2 cm. No major or minor complications occurred in any procedures.

## 4. Discussion

Biopsy and ablation procedures on bone lesions, performed by interventional radiologists under CT-guidance, are widely used and accepted as techniques aimed at the characterization and treatment of primary or metastatic tumors involving the skeletal system [26,27]. However, CT-guidance raises concerns related to the administration of radiation doses to patients, and the lack of real-time control during advancement of instruments inside the patients’ anatomical target regions [28]. Fluoroscopic guidance could represent a solution to track the instrumentation movements in the patient, however it would not solve the issue of patient irradiation and would also add radiation exposure for the operating personnel [29]. In the last years, several guidance systems based on augmented reality navigation have been introduced in different surgical settings [6,30,31], including SIRIO, which has already been validated as an effective tool for the guidance of biopsy procedures on lung lesions [3,23,32,33]. To our knowledge, this is the first study in which an augmented reality navigation system is evaluated as a tool to assist percutaneous procedures on bone lesions in comparison to standard CT-guidance. Our results show a significant reduction in procedural time, number of CT scans and radiation dose in procedures performed under SIRIO guidance. SIRIO’s real time tracking on the biopsy and ablation instruments, in fact, allows to reduce the number of intra-procedural CT acquisitions to assess instrument position with respect to the bone lesion [32]. During the procedures, even with the assistance of SIRIO, intra-procedural scans are usually performed to verify the position and direction of the instrument, but in a much smaller number than those that would be performed with the standard technique. The fewer CT scans result in a lower TDLP value for the patient, which reflects a lower radiation dose for procedures performed with SIRIO. Furthermore, the need for a smaller number of intra-procedural scans shortens the procedural times and improves comfort for the patient, who is required to maintain the same position for the entire duration of the procedure so that a mismatch between the CT acquisition volume and the real patient’s position is not generated. On the contrary, standard CT-guided methods usually provide a higher number of CT scans to check the correct positioning and direction of the instrument, even after small advances or repositioning, weighing on the total radiation dose and on procedural times [34]. Moreover, SIRIO permits avoidance of damage to vulnerable structures near the lesions, such neuro-vascular bundles, allowing a prompt repositioning of the instrument. A higher reduction in all variables was observed for SIRIO-guided procedures compared to standard techniques on lesions smaller than 2 cm (Figure 4). This may reflect the usual inherent ease in reaching large lesions even using standard CT guidance systems which, on the contrary, are less efficient for small lesions for which a high number of intra-procedural CT scans is required without the assistance of active tracking of the used instrument [35,36]. Furthermore, SIRIO guided biopsies showed higher diagnostic accuracy than those performed under standard CT guidance, reflecting a better chance of easily reaching the lesion and taking an adequate sample of tissue for pathological analysis. The reduction of time and resources determined by the use of the navigation system allows to lighten interventional room occupancy and medical and paramedical staff employment, resulting in significant cost savings for the hospital. As with other navigation systems, SIRIO’s correct functioning may be affected by patients’ movements after the first CT-acquisition [6,37]. In those cases, SIRIO will detect the changes and will send an alarm alerting the operator who will be able to decide whether to acquire a new baseline CT to develop the virtual 3D model. Overall, the SIRIO apparatus and mainly the NT and PT are not a hindrance to the instruments and operator movements, allowing a good range of angles and positionings to find the better pathway leading from the skin to the lesion. The greatest issues that preclude the use of SIRIO in routine clinical activity are represented by the unavailability of the system in many centers and by the high level of expertise necessary for its correct use. In general, the use of SIRIO as well as other navigation systems, resulting from technological evolution at the service of medicine and clinical activity, opens up an ever-increasing range of possibilities to ensure greater safety for patients and better optimization of the resources, with potential improvement in the diagnostic–therapeutic management of primary and secondary bone lesions as well as other types of neoplastic pathologies. As demonstrated by the results of our study, the use of SIRIO increases the diagnostic accuracy, especially in small lesions. This depends on the possibility of taking biopsy samples suitable, in terms of quality and quantity, to be subjected to histological and immunohistochemical analyzes which are fundamental for the patient’s prognosis. This is essential in the current concept of precision medicine which, according to a multidisciplinary approach, aims to adapt the treatment to the individual patient in order to optimize clinical efficacy and reduce unwanted effects [38,39,40,41]. This study has some limitations, such as the absence of multi-center involvement and a small sample which we are willing to expand by constantly adding new data. Moreover, procedural times for ablation procedures include the ablation time after the instrument positioning within the lesions, which may differ between RFA, CA and MWA. However, the number of procedures performed using the different techniques are similar between the SIRIO and non-SIRIO groups in both dimensional classes. Interventional radiology procedures on bone represent the ideal field of application for navigation systems by exploiting the intrinsic stability of skeletal structures, unlike other anatomical sites.

## 5. Conclusions

In conclusion, the use of the augmented reality navigation system, SIRIO, significantly improves CT-guided procedures on bone primitive and metastatic lesions, guaranteeing a reduction in patient’s radiation exposure and procedural time. This results in an impact upon the overall procedural quality and resource optimization.

## Figures and Tables

**Table 1:** Groups composition with patients’ characteristics; SD = standard deviation.

Biopsies	SIRIO	Non-SIRIO
<2 cm		
*n* (males; females)	19 (7; 12)	22 (10; 12)
Age (mean) ± SD	61.4 ± 11.9	56.8 ± 13.9
>2 cm		
*n* (males; females)	55 (22; 33)	46 (15; 31)
Age (mean) ± SD	60.9 ± 15.4	61.3 ± 15.3
**Ablations**		
<2 cm		
*n* (males; females)	16 (12; 4)	12 (6; 6)
Age (mean) ± SD	29.4 ± 16.4	29.7 ± 11.1
>2 cm		
*n* (males; females)	11 (5; 6)	12 (5; 7)
Age (mean) ± SD	65.9 ± 8.7	55.5 ± 18.1

**Table 2:** Lesion characteristics including site and histotypes.

Lesion Characteristics	*n*
**Lesion Site**	
Vertebrae	56
Sternum/Ribs	19
Pelvic bones	63
Upper limb bones	12
Lower limb bones	43
**Lesion Types**	
Metastases	100
Osteoid osteomas	31
Multiple myelomas	4
Osteoblastoma	1
Infectious/inflammatory	8
Other non-malignant	35

**Table 3:** Mean values and standard deviations of each variable for all the groups.

Variables	Biopsies				Ablations			
	<2 cm		>2 cm		<2 cm		>2 cm	
	SIRIO	Non-SIRIO	SIRIO	Non-SIRIO	SIRIO	Non-SIRIO	SIRIO	Non-SIRIO
Procedural time (min)	14.6 ± 3	38.1 ± 11.4	12.1 ± 2.8	14 ± 4	33.2 ± 10.7	58.8 ± 9.4	38 ± 10.5	73.6 ± 1.2
CT-s	5 ± 2	9 ± 3	3 ± 2	5 ± 3	5 ± 2	11 ± 2	9 ± 3	14 ± 3
DLP (mGy*cm)	369.8 ± 178.8	829.3 ± 475.1	313.9 ± 132.4	446 ± 371.2	190.3 ± 168.6	360.4 ± 192.3	383.1 ± 117.3	555.8 ± 237.9

CT-s = number of CT-scans; DLP = Dose Length Product.

## Data Availability

The data presented in this study are available on request from the corresponding author. The data are not publicly available due to privacy restrictions.

## References

[1] Berning W., Freyschmidt J., Ostertag H. (1996). Percutaneous bone biopsy, techniques and indications. Eur. Radiol..

[2] Monfardini L., Preda L., Aurilio G., Rizzo S., Bagnardi V., Renne G., Maccagnoni S., Vigna P.D., Davide D., Bellomi M. (2014). CT-guided bone biopsy in cancer patients with suspected bone metastases: Retrospective review of 308 procedures. Radiol. Med..

[3] Grasso R.F., Faiella E., Luppi G., Schena E., Giurazza F., Del Vescovo R., D’Agostino F., Cazzato R.L., Beomonte Zobel B. (2013). Percutaneous lung biopsy: Comparison between an augmented reality CT navigation system and standard CT-guided technique. Int. J. Comput. Assist. Radiol. Surg..

[4] Chehab M.A., Brinjikji W., Copelan A., Venkatesan A.M. (2015). Navigational Tools for Interventional Radiology and Interventional Oncology Applications. Semin. Interv. Radiol..

[5] Wood B.J., Zhang H., Durrani A., Glossop N., Ranjan S., Lindisch D., Levy E., Banovac F., Borgert J., Krueger S. (2005). Navigation with electromagnetic tracking for interventional radiology procedures: A feasibility study. J. Vasc. Interv. Radiol. JVIR.

[6] Appelbaum L., Sosna J., Nissenbaum Y., Benshtein A., Goldberg S.N. (2011). Electromagnetic navigation system for CT-guided biopsy of small lesions. AJR Am. J. Roentgenol..

[7] Bruners P., Penzkofer T., Nagel M., Elfring R., Gronloh N., Schmitz-Rode T., Günther R.W., Mahnken A.H. (2009). Electromagnetic tracking for CT-guided spine interventions: Phantom, ex-vivo and in-vivo results. Eur. Radiol..

[8] Meier-Meitinger M., Nagel M., Kalender W., Bautz W.A., Baum U. (2008). [Computer-assisted navigation system for interventional CT-guided procedures: Results of phantom and clinical studies]. ROFO Fortschr. Geb. Rontgenstr. Nuklearmed..

[9] Aghayev E., Ebert L.C., Christe A., Jackowski C., Rudolph T., Kowal J., Vock P., Thali M.J. (2008). CT data-based navigation for post-mortem biopsy—A feasibility study. J. Forensic Leg. Med..

[10] Khan M.F., Dogan S., Maataoui A., Gurung J., Schiemann M., Ackermann H., Wesarg S., Sakas G., Vogl T.J. (2005). Accuracy of biopsy needle navigation using the Medarpa system--computed tomography reality superimposed on the site of intervention. Eur. Radiol..

[11] Khan M.F., Dogan S., Maataoui A., Wesarg S., Gurung J., Ackermann H., Schiemann M., Wimmer-Greinecker G., Vogl T.J. (2006). Navigation-based needle puncture of a cadaver using a hybrid tracking navigational system. Investig. Radiol..

[12] Zhang Z., Shao G., Zheng J., Wen S., Zeng H., Hao W., Luo J., Guo L. (2020). Electromagnetic navigation to assist with computed tomography-guided thermal ablation of liver tumors. Minim. Invasive Ther. Allied Technol. MITAT Off. J. Soc. Minim. Invasive Ther..

[13] Ringe K.I., Pöhler G.H., Rabeh H., Wacker F. (2021). Electromagnetic Navigation System-Guided Microwave Ablation of Hepatic Tumors: A Matched Cohort Study. Cardiovasc. Interv. Radiol..

[14] Mihalič R., Zdovc J., Mohar J., Trebše R. (2020). Electromagnetic navigation system for acetabular component placement in total hip arthroplasty is more precise and accurate than the freehand technique: A randomized, controlled trial with 84 patients. Acta Orthop..

[15] Attivissimo F., Lanzolla A.M.L., Carlone S., Larizza P., Brunetti G. (2018). A novel electromagnetic tracking system for surgery navigation. Comput. Assist. Surg. Abingdon Engl..

[16] Moulin B., Tselikas L., De Baere T., Varin F., Abed A., Debays L., Bardoulat C., Hakime A., Teriitehau C., Deschamps F. (2020). CT guidance assisted by electromagnetic navigation system for percutaneous fixation by internal cemented screws (FICS). Eur. Radiol..

[17] Liu J.-F., Jiao D.-C., Ren J.-Z., Zhang W.-G., Han X.-W. (2018). Percutaneous bone biopsy using a flat-panel cone beam computed tomography virtual navigation system. Saudi Med. J..

[18] Tselikas L., Joskin J., Roquet F., Farouil G., Dreuil S., Hakimé A., Teriitehau C., Auperin A., de Baere T., Deschamps F. (2015). Percutaneous bone biopsies: Comparison between flat-panel cone-beam CT and CT-scan guidance. Cardiovasc. Interv. Radiol..

[19] Floridi C., Carnevale A., Fumarola E.M., Schampaert S., Fontana F., De Palma D., Del Sole A., Giganti M., Carrafiello G. (2019). Percutaneous Lung Tumor Biopsy Under CBCT Guidance with PET-CT Fusion Imaging: Preliminary Experience. Cardiovasc. Interv. Radiol..

[20] Moser C., Becker J., Deli M., Busch M., Boehme M., Groenemeyer D.H.W. (2013). A novel Laser Navigation System reduces radiation exposure and improves accuracy and workflow of CT-guided spinal interventions: A prospective, randomized, controlled, clinical trial in comparison to conventional freehand puncture. Eur. J. Radiol..

[21] Kagadis G.C., Katsanos K., Karnabatidis D., Loudos G., Nikiforidis G.C., Hendee W.R. (2012). Emerging technologies for image guidance and device navigation in interventional radiology. Med. Phys..

[22] Wu B., Xiao Y.-Y., Zhang X., Zhang A.-L., Li H.-J., Gao D.-F. (2010). Magnetic resonance imaging-guided percutaneous cryoablation of hepatocellular carcinoma in special regions. Hepatobiliary Pancreat. Dis. Int. HBPD INT.

[23] Faiella E., Frauenfelder G., Santucci D., Luppi G., Schena E., Beomonte Zobel B., Grasso R.F. (2018). Percutaneous low-dose CT-guided lung biopsy with an augmented reality navigation system: Validation of the technique on 496 suspected lesions. Clin. Imaging.

[24] Arrigoni F., Bruno F., Zugaro L., Natella R., Cappabianca S., Russo U., Papapietro V.R., Splendiani A., Cesare E.D., Masciocchi C. (2018). Developments in the management of bone metastases with interventional radiology. Acta Bio Med. Atenei Parm..

[25] Huda W., Mettler F.A. (2011). Volume CT dose index and dose-length product displayed during CT: What good are they?. Radiology.

[26] Espinosa L.A., Jamadar D.A., Jacobson J.A., DeMaeseneer M.O., Ebrahim F.S., Sabb B.J., Kretschmer M.T., Biermann J.S., Kim S.-M. (2008). CT-guided biopsy of bone: A radiologist’s perspective. AJR Am. J. Roentgenol..

[27] Foster R.C.B., Stavas J.M. (2014). Bone and Soft Tissue Ablation. Semin. Interv. Radiol..

[28] Guberina N., Forsting M., Ringelstein A., Suntharalingam S., Nassenstein K., Theysohn J., Wetter A. (2018). Radiation exposure during CT-guided biopsies: Recent CT machines provide markedly lower doses. Eur. Radiol..

[29] Kurban L.A., Gomersall L., Weir J., Wade P. (2008). Fluoroscopy-guided percutaneous lung biopsy: A valuable alternative to computed tomography. Acta Radiol..

[30] Laine T., Lund T., Ylikoski M., Lohikoski J., Schlenzka D. (2000). Accuracy of pedicle screw insertion with and without computer assistance: A randomised controlled clinical study in 100 consecutive patients. Eur. Spine J. Off. Publ. Eur. Spine Soc. Eur. Spinal Deform. Soc. Eur. Sect. Cerv. Spine Res. Soc..

[31] Bolger C., Wigfield C. (2000). Image-guided surgery: Applications to the cervical and thoracic spine and a review of the first 120 procedures. J. Neurosurg..

[32] Iannelli G., Caivano R., Villonio A., Semeraro V., Lucarelli N.M., Ganimede M.P., Gisone V., Dinardo G., Bruno S., Macarini L. (2018). Percutaneous Computed Tomography-Guided Lung Biopsies using a Virtual Navigation Guidance: Our Experience. Cancer Investig..

[33] Grasso R.F., Cazzato R.L., Luppi G., D’Agostino F., Schena E., Del Vescovo R., Giurazza F., Faiella E., Beomonte Zobel B. (2013). Percutaneous lung biopsies: Performance of an optical CT-based navigation system with a low-dose protocol. Eur. Radiol..

[34] Weir V.J., Zhang J., Bruner A.P. (2014). Impact of physician practice on patient radiation dose during CT guided biopsy procedures. J. X-ray Sci. Technol..

[35] Huang M.-D., Weng H.-H., Hsu S.-L., Hsu L.-S., Lin W.-M., Chen C.-W., Tsai Y.-H. (2019). Accuracy and complications of CT-guided pulmonary core biopsy in small nodules: A single-center experience. Cancer Imaging.

[36] Li Y., Du Y., Yang H.F., Yu J.H., Xu X.X. (2013). CT-guided percutaneous core needle biopsy for small (≤ 20 mm) pulmonary lesions. Clin. Radiol..

[37] Zhou Y., Thiruvalluvan K., Krzeminski L., Moore W.H., Xu Z., Liang Z. (2013). CT-guided robotic needle biopsy of lung nodules with respiratory motion—Experimental system and preliminary test. Int. J. Med. Robot. Comput. Assist. Surg. MRCAS.

[38] Johnson T.M. (2017). Perspective on Precision Medicine in Oncology. Pharmacotherapy.

[39] König I.R., Fuchs O., Hansen G., von Mutius E., Kopp M.V. (2017). What is precision medicine?. Eur. Respir. J..

[40] Ramaswami R., Bayer R., Galea S. (2018). Precision Medicine from a Public Health Perspective. Annu. Rev. Public Health.

[41] Weil A.R. (2018). Precision Medicine. Health Aff. Proj. Hope.

